# Block of proliferation 1 promotes cell migration and invasion in human colorectal cancer cells via the JNK pathway

**DOI:** 10.1002/jcla.23283

**Published:** 2020-03-13

**Authors:** Xiaoxi Chen, Yu Zhao

**Affiliations:** ^1^ Department of Gastroenterology Affiliated Maternity and Child Health Care Hospital of Nantong University Nantong China; ^2^ Qingdao Hospital of Traditional Chinese Medicine (Qingdao Hiser Hospital) Qingdao China

**Keywords:** block of proliferation 1, colorectal cancer, invasion, JNK signaling, migration

## Abstract

Metastasis is one of the most common causes of death in patients with colorectal cancer (CRC). Block of proliferation 1 (BOP1) regulates tumorigenesis, epithelial‐to‐mesenchymal transition, migration, metastasis, and drug resistance in several tumor types. However, the role of BOP1 in the regulation of colorectal cancer cell migration and invasion is still largely unclear. In this study, the results of immunohistochemistry showed that BOP1 was upregulated in our cohort of CRC patients. BOP1 knockdown inhibited the migration and invasion of CRC cells, confirmed by the downregulation of the mRNA levels of MMP‐2 and MMP‐9. The overexpression of BOP1 in CRC cells exerted the opposite effect. SP600125, an inhibitor of JNK signaling, partially abolished the BOP1 overexpression‐mediated increase in the migratory and invasive ability of CRC cells. Our results indicated that BOP1 is an important regulator of CRC cell invasion and migration, predominantly through the JNK signaling pathway.

## 1 INTRODUCTION

Colorectal cancer (CRC) is one of the most common gastrointestinal tumors and occurs in the colon or rectum.^1^, ^2^, ^3^ Metastasis has been reported as the most common cause of death in patients with CRC.^4^, ^5^, ^6^ The development and progression of CRC involve a large number of signaling pathways and oncogenes^7^, ^8^; however, the mechanisms underlying CRC metastasis remain largely unknown. Therefore, research on novel molecular processes that control CRC metastasis may provide effective therapies for the treatment of CRC.

Block of proliferation 1 (BOP1), which contains WD40 repeats, is one part of the PES1‐BOP1‐WDR12 (PeBoW) complex involved in ribosomal RNA processing.^9^ More recently, BOP1 was reported to be involved in various malignancies. In recent years, multiple studies have shown that BOP1 regulates tumorigenesis, epithelial‐to‐mesenchymal transition, migration, metastasis, and drug resistance in several tumor types, such as colorectal cancer,^10^ hepatocellular carcinoma,^11^ and melanoma.^12^ So far, the study of BOP1 in cancers has been limited, and comprehensive data are not available. Hence, it is necessary to determine the role of BOP1 in tumor progression, especially in migration and invasion of cancer.

Here, BOP1 was determined to be highly overexpressed in a cohort of patients with CRC. BOP1 had different roles in metastatic CRC cells. Moreover, BOP1 promoted CRC cell migration and invasion by regulating the JNK pathway. Therefore, our results suggest that BOP1 is an important regulator of CRC cell invasion and migration, predominantly through the JNK signaling pathway, and raise the possibility of using this molecule as an indicator for CRC treatment.

## 2 MATERIALS AND METHODS

### 2.1 Clinical specimens

Forty‐six pairs of CRC tissue samples from patients who underwent surgery and were pathologically diagnosed at Nantong University Maternal and Child Health Hospital were recruited. None of the CRC patients in this study received preoperative chemotherapy or radiotherapy. The Union for International Cancer Control classification was used to determine the clinical stage. The study protocol was approved by the Ethics Committee of Nantong University Maternal and Child Health Hospital. Written informed consent procedures were obtained from each patient.

### 2.2 Immunohistochemistry

Immunohistochemistry (IHC) for BOP1 protein expression was performed as previously described.^13^, ^14^ Briefly, 5 μm thick paraffin‐embedded tissue sections were deparaffinized, hydrated, rinsed, and subjected to antigen retrieval. Then, the tissue sections were incubated with 3% bovine serum albumin (BSA; Beyotime) and then incubated with anti‐BOP1 antibody (Invitrogen) overnight at 4°C. After incubation with the corresponding HRP‐conjugated secondary antibody, BOP1 expression was detected by 3,3N‐diaminobenzidine tetrahydrochloride (DAB; Sangon Biotech). The BOP1 expression levels were evaluated under a light microscope (Olympus) and scored as follows: 0, 5% or less positive cells; 1, 5%‐25% positive cells; 2, 26%‐75% positive cells; and 3, 76% and higher positive cells. Stain intensity was scored as follows: 0, no staining; 1, faint yellow; 2, brown‐yellow; and 3, dark brown. All IHC slides were reviewed independently by two investigators.

### 2.3 Cell culture

Colorectal cancer cell lines SW480, SW620, CaCO2, HCT116, and HT29 were obtained from the American Type Culture Collection (Manassas). SW480 and SW620 cells were cultured in RPMI‐1640 medium (HyClone). CaCO2, HCT116, and HT29 cells were maintained in Dulbecco's modified Eagle's medium (HyClone). Ten percent fetal bovine serum (FBS; HyClone) was added to all media.

### 2.4 Cell transfection

Two BOP1 siRNAs and scramble siRNA were purchased from GeneChem Co., Ltd. The vector pcDNA3.1 containing the full‐length BOP1 cDNA sequence (BOP1) and control vector (vector) were obtained from GeneCopoeia Inc. siRNA or plasmid transient transfections were performed using Lipofectamine 2000 (Invitrogen) according to the recommended instructions.

### 2.5 Western blot analysis

Western blot for BOP1, P‐JNK, and JNK protein expression was performed as previously described.^15^, ^16^ Briefly, 30 μg of total protein was separated by 10% SDS‐PAGE and transferred onto a nitrocellulose membrane (Bio‐Rad). Anti‐human BOP1 (Invitrogen), anti‐human P‐JNK (Abcam), and anti‐human JNK antibodies (Abcam) were incubated with the membranes overnight at 4°C. Then, the membranes were incubated with HRP‐conjugated secondary antibodies (CST) and visualized with a chemiluminescent detection system (Beyotime). Protein levels were quantified by density analysis using ImageJ software (National Institutes of Health).

### 2.6 RNA extraction and quantitative real‐time polymerase chain reaction

Total RNA was extracted from cultured cells using TRIzol reagent (Invitrogen) according to the manufacturer's protocol. To detect BOP1 expression, 1 μg of total RNA was synthesized into complementary DNA using the PrimeScript RT reagent kit (TaKaRa). QRT‐PCR was performed using SYBR Premix Ex Taq II (TaKaRa). Expression was normalized to glyceraldehyde 3‐phosphate dehydrogenase (GAPDH). The primer sequences are as follows: BOP1 forward: 5′‐CTGGTTTCCATCCCCAGTTG‐3′, BOP1 reverse: 5′‐GGTTGCCCGAGCATCGT‐3′; GAPDH forward: 5′‐CTGGGCTACACTGAGCACC‐3′, and GAPDH reverse: 5′‐AAGTGGTCGTTGAGGGCAATG‐3′.

### 2.7 Migration and invasion assays

The cell migration and invasion assays were performed as previously described.^17^, ^18^ For the migration assay, HT29 or SW480 cells in serum‐free medium were seeded into a 24‐well Insert System with an 8‐μm pore size polyethylene terephthalate membrane (BD Biosciences). Complete medium was placed in the lower chambers. For the cell invasion assay, HT29 or SW480 cells in serum‐free medium were seeded into the chambers precoated with a matrix gel (BD Biosciences). Cells that had migrated or invaded through the membrane were fixed using 10% methanol, stained with 0.1% crystal violet, and quantified using a light microscope (Olympus). Migrated and invasive cells were counted from 5 randomly selected fields of each sample.

### 2.8 Statistical analysis

All results in this study are presented as the mean ± SEM. Statistical analysis was performed using GraphPad Prism 6.0 software by Student's *t* test. For clinical data analysis, the correlation between 2 groups was analyzed by the Spearman correlation test. A *P*‐value <.05, .01, or .001 was considered significant.

## 3 RESULTS

### 3.1 BOP1 protein expression in CRC tissues and correlation between BOP1 and clinicopathological features of patients with CRC

**Figure 1:**
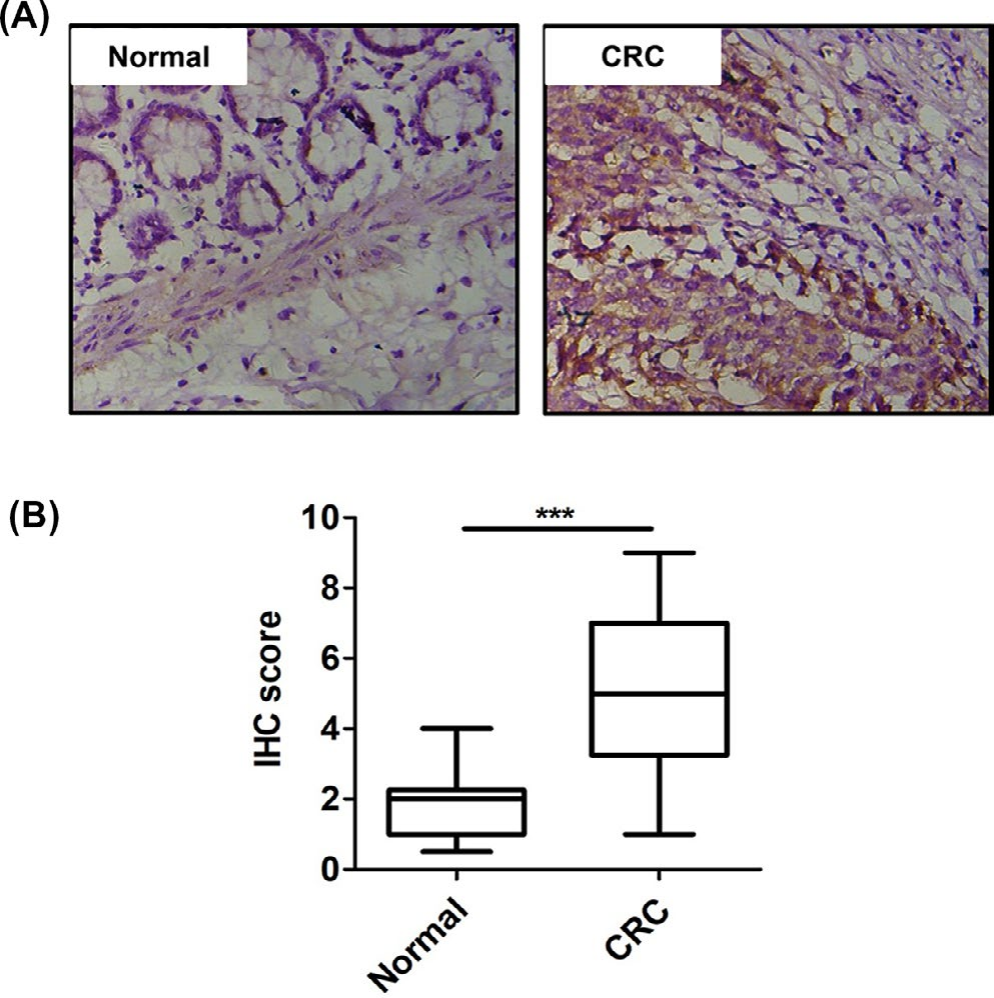
Block of proliferation 1 (BOP1) was upregulated in tissue samples of patients with CRC. A, Representative IHC staining image of BOP1 in CRC tissues. B, Quantitation of BOP1‐positive tumor cells

To examine BOP1 protein expression in CRC tissues, we performed IHC analysis on both tumor tissue samples and matched adjacent nontumor tissue samples. Compared with that in paired adjacent normal tissue samples, BOP1 expression was significantly upregulated in tumor tissues (Figure 1A and 1B). Furthermore, we analyzed the association between the protein level of BOP1 and the clinicopathological features of CRC patients. BOP1 expression in tissues of CRC patients was associated with TNM stage, lymph node metastasis, and distant metastasis (Table 1, *P* < .05). In addition, there was no association between BOP1 expression in tissues of CRC patients and age, gender, or tumor size. These clinical results showed that BOP1 overexpression in CRC patients might be associated with CRC aggressiveness, especially tumor metastasis.

**Table 1:** Association between block of proliferation 1 (BOP1) expression and clinicopathological features

Clinicopathological Features	N	BOP1 Expression		*P* Value
		Low	High	
Age (y)				
≤60	28	13	15	.162
>60	18	7	11	
Gender				
Female	20	11	9	.257
Male	26	10	16	
Tumor size (cm)				
≤2	17	12	5	.342
>2	29	16	13	
TNM stage				
I + II	10	8	2	.037*
III + IV	36	12	24	
Lymph node metastasis				
Positive	27	5	22	.021*
Negative	19	12	7	
Distant metastasis				
Positive	34	7	27	.041*
Negative	12	8	4	

Abbreviation: TNM, tumor node metastasis.

* Statistically significant (*P < .05).

### 3.2 BOP1 knockdown inhibits the migration and invasion of HT29 cells

**Figure 2:**
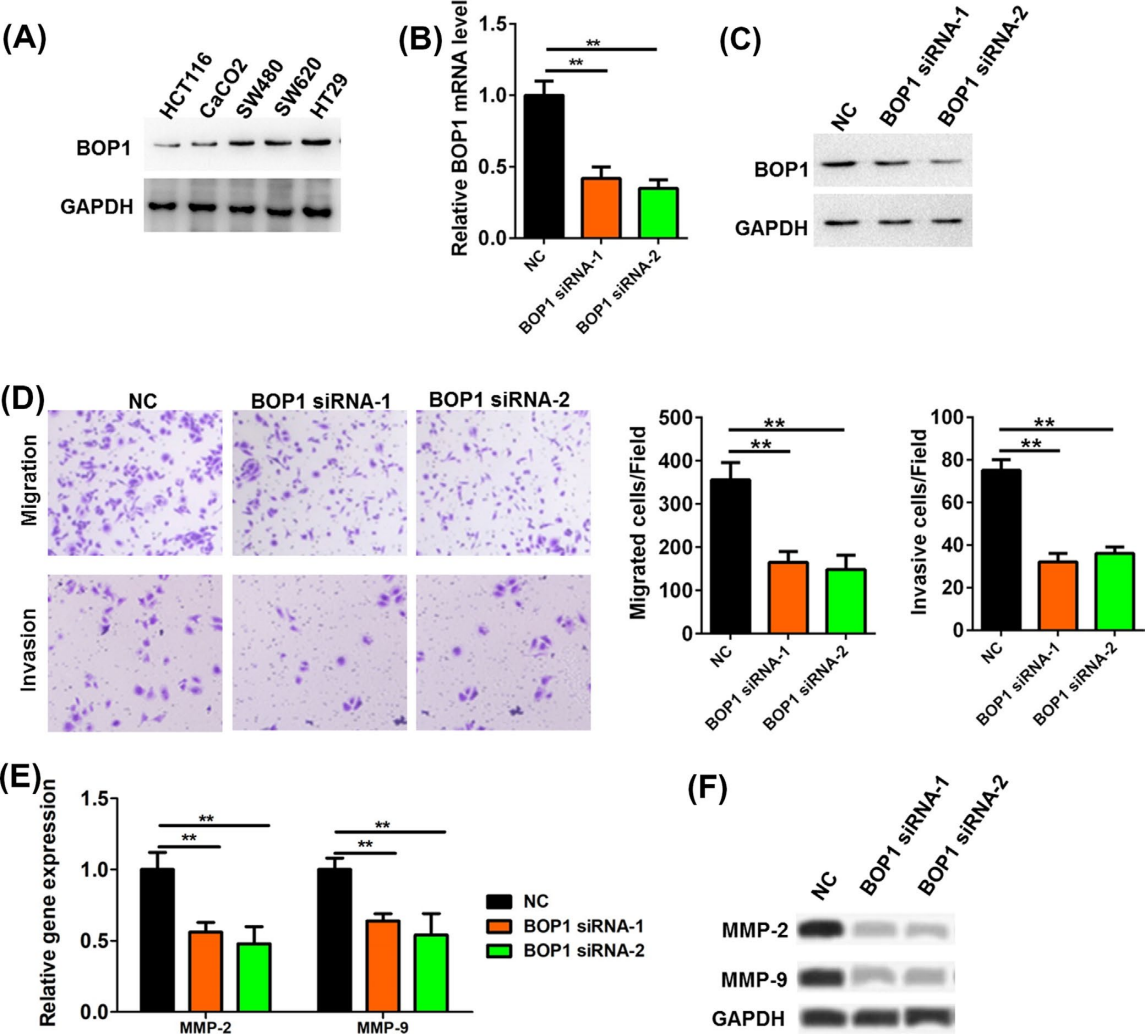
Block of proliferation 1 (BOP1) knockdown inhibited the migration and invasion of HT29 cell. A, The protein level of BOP1 in multiple CRC cell lines (HCT116, CaCO2, SW480, SW620, and HT29) with different metastatic potential was analyzed by Western blot. B, C, The mRNA (B) and protein (C) level of BOP1 in HT29 cells after transfected with BOP1 siRNAs. D, The cell migrated and invasive activity of HT29 cells was investigated when HT29 cells were transfected with BOP1 siRNAs. E, F, The mRNA (E) and protein (F) level of MMP‐2 and MMP‐9 in HT29 cells after transfected with BOP1 siRNAs. **P < .01

The protein levels of BOP1 in multiple CRC cell lines with different metastatic potential were analyzed by Western blot. The level of BOP1 was higher in the highly metastatic CRC cell lines (HT29 and SW620) than in the low‐metastatic CRC cell lines (HCT116 and CaCO2) (Figure 2A). The BOP1 expression level was highest in HT29 cells (Figure 2A). Two specific BOP1 siRNAs significantly downregulated both the mRNA and protein levels of BOP1 in HT29 cells (Figure 2B and 2C). BOP1 siRNAs could decrease the migratory and invasive activity of HT29 cell (Figure 2D). MMP‐2 and MMP‐9 have been reported to be involved in metastasis processes of CRC.^19^, ^20^ Herein, we used these two proteins as metastasis markers to evaluate the effect of BOP1 on the migration and invasion of CRC cells. We observed that BOP1 knockdown downregulated the mRNA and protein levels of MMP‐2 and MMP‐9 (Figure 2E and 2F).

### 3.3 BOP1 overexpression promotes the migration and invasion of HCT116 cells

**Figure 3:**
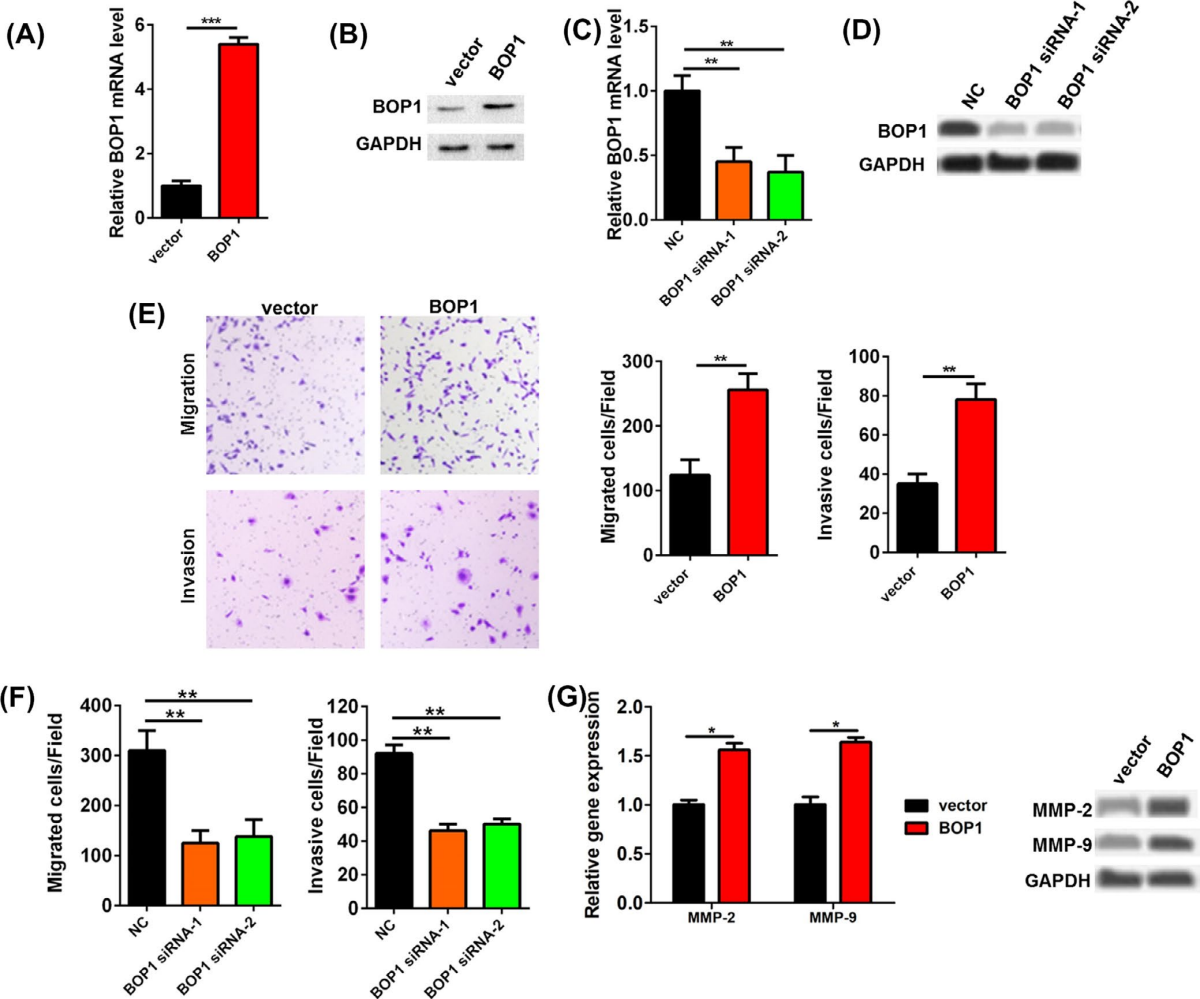
Block of proliferation 1 (BOP1) promoted the migration and invasion of HCT116 cell. (A, B) The mRNA (A) and protein (B) level of BOP1 in HCT116 cells after transfected with pcDNA3.1‐BOP1 (BOP1) plasmids. C, D, The mRNA (C) and protein (D) level of BOP1 in HCT116 cells after transfected with BOP1 siRNAs. E, The cell migrated and invasive activity of HCT116 cells was investigated when HCT116 cells were transfected with BOP1 plasmids. F, The cell migrated and invasive activity of HCT116 cells was investigated when HCT116 cells were transfected with BOP1 siRNAs. G, The mRNA and protein level of MMP‐2 and MMP‐9 in HCT116 cells after transfected with BOP1 plasmids. *P < .05, **P < .01, ***P < .001

The pcDNA3.1 vector containing BOP1 cDNA (BOP1) or the control vector (vector) was transfected into HCT116 cells. The BOP1 vector significantly upregulated the mRNA and protein levels of BOP1 in HCT116 cells (Figure 3A and 3B). In addition, both BOP1 siRNA‐1 and BOP1 siRNA‐2 significantly downregulated both the mRNA and protein levels of BOP1 in HCT116 cells (Figure 3C and 3D). The BOP1 vector could increase the migratory and invasive activity of HCT116 cells (Figure 3E). In contrast, BOP1 siRNAs could decrease the migratory and invasive activity of HCT116 cells (Figure 3F). Moreover, BOP1 overexpression upregulated the mRNA and protein levels of MMP‐2 and MMP‐9 (Figure 3G).

### 3.4 BOP1 knockdown inhibits the JNK signaling pathway

**Figure 4:**
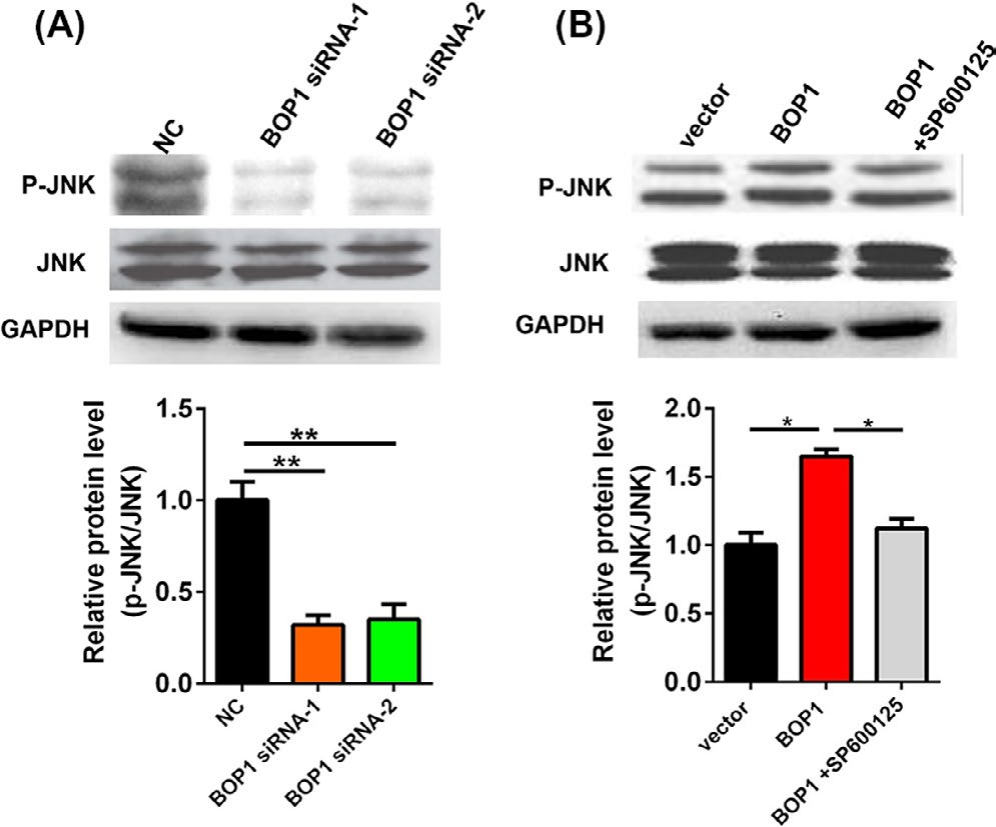
Block of proliferation 1 (BOP1) knockdown inhibits JNK signaling pathway. A, The protein level of p‐JNK and JNK in HT29 cells after transfected with BOP1 siRNAs. B, The protein level of p‐JNK and JNK in HCT116 cells after transfected with BOP1 plasmids. *P < .05, **P < .01

A previous study reported that BOP1 could activate the JNK signaling pathway and further affect migration in CRC.^10^ Hence, we hypothesized that BOP1 knockdown may reduce the migratory and invasive activity of CRC cells via the JNK signaling pathway. As shown in Figure 4A, Western blot analysis indicated that the p‐JNK level in HT29 cells transfected with the two specific BOP1 siRNAs was significantly decreased. In contrast, the p‐JNK level in HCT116 cells transfected with the BOP1 vector was significantly increased (Figure 4B). Moreover, SP600125, a JNK signaling pathway inhibitor, decreased the p‐JNK level induced by BOP1 overexpression (Figure 4B).

### 3.5 JNK signaling inhibitor reduces BOP1 overexpression‐induced migration and invasion of HCT116 cells

**Figure 5:**
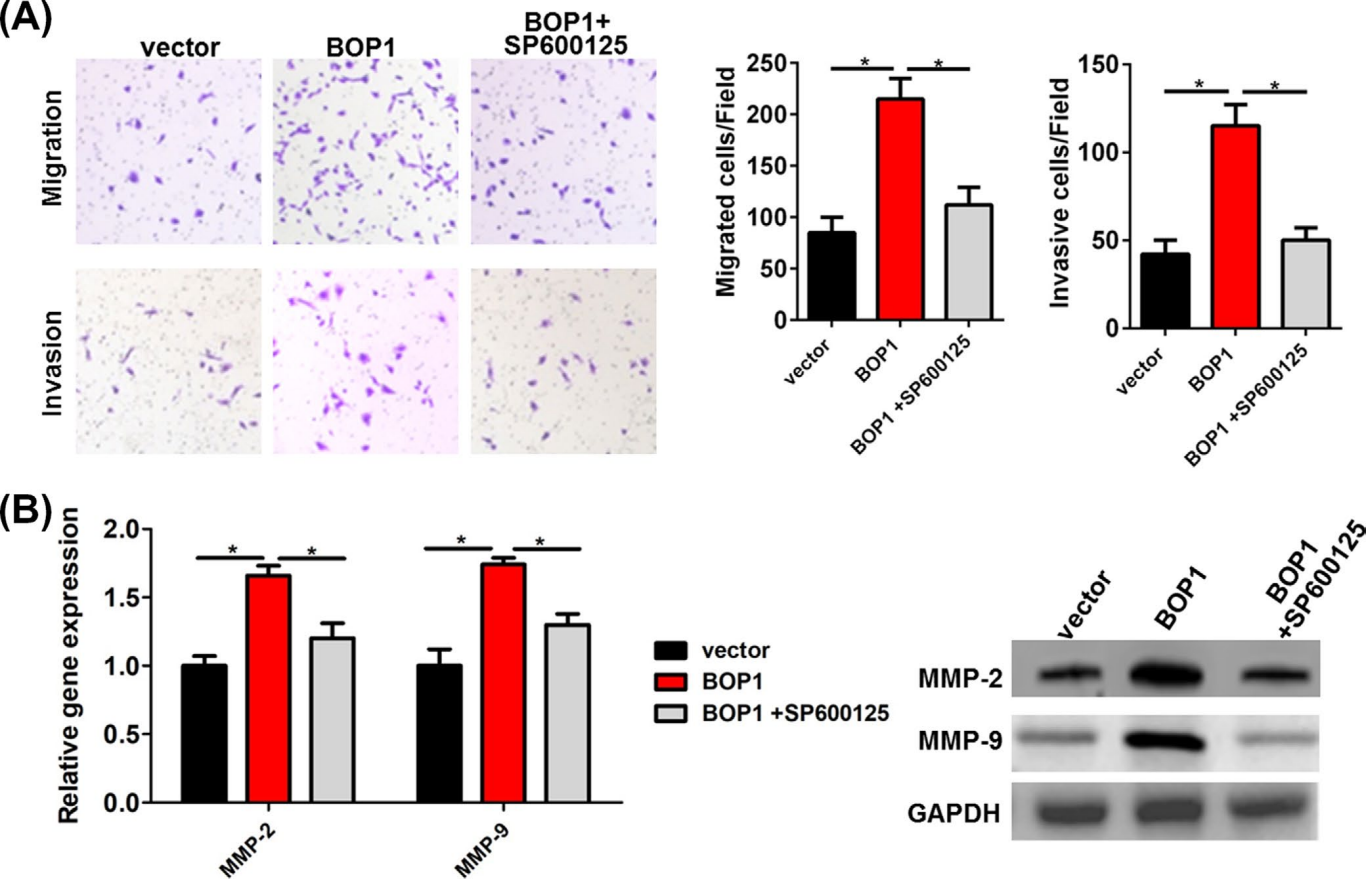
Inhibition of JNK signaling reduced block of proliferation 1 (BOP1) overexpression‐induced migration and invasion of HCT116 cell. A, Effect of BOP1 overexpression on the cell migrated and invasive activity of HCT116 cells was abolished by the JNK signaling inhibitor SP600125. B, The mRNA and protein level of MMP‐2 and MMP‐9 in HCT116 cells co‐treated with BOP1 plasmids and SP600125. The increased expression of MMP‐2 and MMP‐9 in HCT116 cells after transfected with BOP1 plasmids was abolished by the JNK signaling inhibitor SP600125. *P < .05

To further confirm that JNK signaling was involved in BOP1 overexpression‐induced migration and invasion, HCT116 cells transfected with the BOP1 vector were treated with the JNK signaling inhibitor SP600125. HCT116 cells transfected with the BOP1 vector showed higher migratory and invasive activity than those transfected with the control vector, whereas SP600125 reversed the BOP1 overexpression‐mediated increase in the migratory and invasive ability of HCT116 cells (Figure 5A). Moreover, SP600125 reversed the BOP1 overexpression‐mediated increase in MMP‐2 and MMP‐9 mRNA and protein levels in HCT116 cells (Figure 5B).

## 4 DISCUSSION

Multiple genetic changes in tumor suppressor genes and oncogenes are involved in the progression of CRC.^21^, ^22^, ^23^ A previous study showed that abnormal BOP1 expression was observed in hepatocellular carcinoma clinical specimens.^11^ In the present study, we performed IHC analysis of both tumor tissue samples and matched adjacent nontumor tissue samples to analyze the expression level of BOP1 in patients with CRC. BOP1 expression was significantly upregulated in CRC tumor tissues (Figure 1). Furthermore, BOP1 expression in the tissues of CRC patients was associated with TNM stage, lymph node metastasis, and distant metastasis (Table 1, *P* < .05). In addition, there was no association between BOP1 expression in tissues of CRC patients and age, gender, or tumor size. Overall, BOP1 overexpression in CRC patients might be associated with CRC aggressiveness, especially tumor metastasis.

BOP1 has been reported to participate in various tumor biological processes, such as tumorigenesis, epithelial‐to‐mesenchymal transition, migration, and drug resistance.^10^, ^11^, ^12^ Qi et al reported that BOP1 is an important target gene of the Wnt/β‐catenin pathway and is involved in the Wnt/β‐catenin pathway‐induced epithelial‐to‐mesenchymal transition, cell migration, and experimental metastasis of CRC cells.^10^ Chung et al found that BOP1 was upregulated in tumors compared with adjacent nontumoral liver specimens.^11^ BOP1 knockdown clearly upregulated epithelial markers (E‐cadherin, cytokeratin 18, and γ‐catenin) and downregulated mesenchymal markers (fibronectin and vimentin).^11^ BOP1 knockdown resulted in resistance to BRAF kinase inhibitors both in melanoma cell culture and in a melanoma mouse model.^12^ In the present study, we found that BOP1 knockdown in the highly metastatic CRC HT29 cells could decrease the migratory and invasive activity of these cells, while BOP1 overexpression in the low‐metastatic HCT116 CRC cells could increase the migratory and invasive activity of the cells. Hence, these results indicated that BOP1 may participate in the invasion and migration of CRC.

Previous studies have indicated that BOP1 can regulate tumor development by various known regulatory pathways.^10^, ^11^, ^12^, ^24^ It has been reported that BOP1 knockdown resulted in the downregulation of the MAPK phosphatases DUSP4 and DUSP6 and further increased MAPK signaling and resistance to BRAF kinase inhibitors in melanoma.^12^ BOP1 could promote the epithelial‐to‐mesenchymal transition by stimulating actin stress fiber assembly and RhoA activation in hepatocellular carcinoma.^11^ Moreover, BOP1 was found to be involved in the Wnt/β‐catenin/JNK signaling pathway‐mediated experimental metastasis and migration of CRC cells.^10^ Therefore, we assumed that BOP1 could promote CRC cell invasion and migration through the JNK signaling pathway. BOP1 knockdown significantly decreased the p‐JNK level in HT29 cells, while BOP1 overexpression significantly increased the p‐JNK level in HCT116 cells. Moreover, SP600125 decreased the p‐JNK level induced by BOP1 overexpression. It is important that SP600125 reversed the BOP1 overexpression‐mediated increase in the migratory and invasive ability of HCT116 cells. Overall, BOP1 promoted the invasion and migration of CRC cells through the JNK signaling pathway.

In conclusion, our results indicated that BOP1 is a key regulator of CRC cell invasion and migration, predominantly through the JNK signaling pathway. BOP1 may be a key component of the JNK prometastatic signaling network in CRC cells.

## AUTHOR CONTRIBUTIONS

Xiaoxi Chen conceived and designed the experiments and provided experimental guidance in the laboratory; Yu Zhao analyzed the data and wrote the article.
